# Open Joints*Read before Annual Meeting of the Pennsylvania State Veterinary Medical Association, March 8th, 1893.

**Published:** 1893-03

**Authors:** J. C. Michener


### OPEN JOINTS.*

By Dr. J. C. Michener.

This subject is chosen for the purpose of giving a special treatment. The anatomical structure and the various injuries and operations by which synovial capsules are opened, or become so, by

* Read before Annual Meeting of the Pennsylvania State Veterinary Medical Association, March 8th, 1893.

the suppurative process, will be alluded to only as deemed necessary to illustrate the principle of treatment.

In the management of the larger animals we encounter a great difficulty, in as much as we cannot enforce that repose which the human surgeon finds so necessary in the treatment of open joints.

Our cases, if left to the unaided powers of nature, result in anchylosis or a suppurative arthritis and the destruction of the patient's life. These results, coupled with the intense suffering, make it a matter of great import to devise means by which a prompt and complete cure may be effected.

The first indication is to close the wound to prevent the escape of synovia and the ingress of deleterious germs ; secondly, to combat the tendency to inflammation, more or less severe from the amount of laceration, contusion and lapse of time that the joint has lain open.

The chief obstacle to permanently closing an open joint is the very slow process of repair in ligamentous coverings. Ligament is devoid of sensibility and a complete circulation, giving low vitality.

The breach of continuity is first closed by the product of osmosis, being organized into a thin, fibrous film, requiring several weeks to harden and thicken into true ligament. In the usual course it proves too delicate to withstand the tension of the increased synovial secretion, and the difficulty is further increased by a constant tendency to suppuration.

Now, without decrying the usefulness of the usual remedies, such as the application of styptic colloid, shellac or collodion, to exclude the air and irrigation, to keep down inflammation, we have a very much superior treatment to offer. One that better fills the indications and proves highly satisfactory in practice.

Simply the application of good antiseptic blisters, supplemented by such surgical aids as the nature of injury requires.

The most common accident to joints met in the country is puncture by frost nail, calk of shoe or tine of fork ; such injuries at first appearing very trivial to the inexperienced, requiring from three to ten days to slough a free opening and establish active synovitis, consequently valuable time is lost and a very easily managed affair becomes one of serious import.

In all such cases, when seen before much discharge occurs or lameness supervenes, should have all inverted hairs, shreds of skin or dirt carefully removed, and the hair clipped over a space the size of a silver dollar, or over the entire joint in severe cases.

Now, the blister ointment is liberally applied and well rubbed in, filling up the wound and coating it over. This should be watched for two days and reapplied as it melts from the opening or fails to act over the clipped surface. Keep up the counter-irritation for three days and your patient is safe and ready to go to work as soon as the effect of the blister subsides.

If the case has been neglected until there is great inflammation, pain and lameness, and the synovia is sent out in jets at each movement, but has not yet become bloody, we proceed as before, preparing for an external blister over the entire joint, and in addition provide ourselves with a portion of melted ointment, a syringe with nozzle that nicely fills the opening, a plug of fine cotton, pieces of muslin, thickly coated with the ointment to envelop the joint, and bandages.

After twitching the patient and using an injection of cocaine, proceed to inject the joint full as possible with the melted blister and plug quickly and retain with a suture if necessary. Apply the external dressings and allow them to remain twelve hours when they are removed, the plug taken out, and a rag, covered thickly with the ointment, placed over the wound and remainder of the joint, if the first application has failed to act thoroughly.

In twenty-four hours the suffering patient that stood on three legs, with the lame one in almost constant motion, will have its weight upon the injured limb, resting the opposite, breathing easily and taking his feed.

In rare severe cases the pain may recur in a few days and the wound point and reopen, when the same treatment is repeated. If by any mishap the case has become chronic, repeated blisters and irrigation during the intervals is the best I have found.

Where we have extensive laceration, ligament torn through, tendon off and fracture of bone, I have plastered the wound until closed as a mason would a crack in a wall, brought the parts together with sutures, and kept the wound and whole injured part covered with blister with the most happy results.

In short, whenever any of the hard tissues of the limbs, joints or feet are wounded, my practice is to get blister ointment in and around the part quickly as possible. It draws more blood to the part, causes the opening to swell shut, acts as a bandage, lessens motion, and the process of repair goes on rapidly, while the counter-irritation preserves the internal parts from a destructive inflammation, ending in suppuration, exostosis and anchylosis.

This treatment is not new with our family and I apply it with the greatest confidence in all cases, except where the discharge has become ichorous and bloody, indicating exfoliation of the ends of the bones, which, if in an important joint, renders the case hopeless.

The blister must be antiseptic. Simply cantharides rubbed into stale lard will not answer. The body of the ointment must consist of beeswax, rosin and lard in proportions to suit the season, to every pound of which should be added four ounces of spirits of turpentine in which sulphuric acid has been slowly stirred until effervescence has taken place, cantharides four ounces.

The patient should be early in the slings and may have such internal remedies as his condition indicates.

