# Actinomycosis Bovis, or “Lump Jaw”

**Published:** 1893-11

**Authors:** N. S. Mayo

**Affiliations:** Experiment Station, Kansas State Agricultural College


### CORRECTION.

### ACTINOMYCOSIS BOVIS, OR "LUMP JAW."

By Prof. N. S. Mayo, D.V.S.,
*Experiment Station, Kansas State Agricultural College.*

In the March number, Vol. XIV, No. 3, fol. 163, of this Journal, the article on "Actinomycosis Bovis, or 'Lump Jaw,'" was improperly attributed to R. R. Dinwiddie, V.S. The error occurred in the Editorial Office; the copy did not have Dr. Mayo's name and had been filed with a letter of Dr. Dinwiddie's relative to the subject. We offer Dr. Mayo our apologies and shall claim from him another article to prove our appreciation, and Dr. Dinwiddie owes one in payment of his temporary colors.

